# ^15^N Natural Abundance Evidences a Better Use of N Sources by Late Nitrogen Application in Bread Wheat

**DOI:** 10.3389/fpls.2018.00853

**Published:** 2018-06-22

**Authors:** Teresa Fuertes-Mendizábal, José M. Estavillo, Miren K. Duñabeitia, Ximena Huérfano, Ander Castellón, Carmen González-Murua, Ana Aizpurua, María Begoña González-Moro

**Affiliations:** ^1^Department of Plant Biology and Ecology, University of the Basque Country (UPV/EHU), Bilbao, Spain; ^2^NEIKER-Tecnalia, Basque Institute for Agricultural Research and Development, Derio, Spain

**Keywords:** bread quality, bread wheat, humid Mediterranean conditions, ^15^N natural abundance, *Triticum aestivum*

## Abstract

This work explores whether the natural abundance of N isotopes technique could be used to understand the movement of N within the plant during vegetative and grain filling phases in wheat crop (*Triticum aestivum* L.) under different fertilizer management strategies. We focus on the effect of splitting the same N dose through a third late amendment at flag leaf stage (GS37) under humid Mediterranean conditions, where high spring precipitations can guarantee the incorporation of the lately applied N to the soil-plant system in an efficient way. The results are discussed in the context of agronomic parameters as N content, grain yield and quality, and show that further splitting the same N dose improves the wheat quality and induces a better nitrogen use efficiency. The nitrogen isotopic natural abundance technique shows that N remobilization is a discriminating process that leads to an impoverishment in ^15^N of senescent leaves and grain itself. This technique also reflects the more efficient use of N resources (fertilizer and native soil-N) when plants receive a late N amendment.

## Introduction

Nitrogen is one of the essential nutrients required for crop productivity, which is mainly provided in form of fertilizers. However, the overuse of fertilizers in nowadays agriculture is often associated with a low nitrogen use efficiency (NUE) by the crop, since most crop plants absorb only 30–50% of the N fertilizer supplied, depending on the soil or the environment (Tilman et al., 2002; Ladha et al., 2005). This N overapplication of fertilizer derives in a risk of negative impacts to the environment.

During the last decades, innovative approaches such as quantitative genetics or different agronomic strategies are being introduced in order to increase crops NUE. Among agronomic practices, an adequate distribution of the fertilizer rate is fundamental in order to assure that the N applied is available at the right time, especially in early growth stages. In wheat crop, two amendments of N fertilizer are traditionally applied in Mediterranean areas. The first and second amendments are applied, according to Zadoks growth scale for cereals, at tillering stage (GS20) and at stem elongation stage, respectively (Zadoks et al., 1974). However, Gate (1995) and Oury et al. (2003) proposed the application of a third amendment in the flag leaf stage (GS37), which would extend the vegetative growth of the plant and enhance the N content of the vegetative organs. This would lead to a higher N availability in the vegetative organs to be later remobilized within the plant at the reproductive stage, eventually improving grain protein concentration. This fertilizer management strategy is commonly used in European regions of the United Kingdom, Germany, Netherlands, or Denmark, where high spring rains result in higher availability of N fertilizer to crop plants. Nevertheless, under Mediterranean climatic conditions a late N application has led to very different results regarding grain yield (Alcoz et al., 1993; Garrido-Lestache et al., 2005; Ercoli et al., 2013; Blandino et al., 2015) and grain quality in terms of N concentration (Ayoub et al., 1994; Garrido-Lestache et al., 2005; Brown and Petrie, 2006; Fuertes-Mendizábal et al., 2010a; Blandino et al., 2015). These contrasting results are probably explained by the different climatic conditions under which field experiments have been carried out.

In the Basque Country cereal production is conducted under humid Mediterranean conditions. Under these climatologic conditions a late N amendment at GS37 could be relevant due to high spring precipitations can lead to higher grain N content, as a result of an efficient incorporation of the lately applied N (Fuertes-Mendizábal et al., 2010a), in contrast to other drier Mediterranean areas. In fact, under irrigated conditions wheat plants grown in pots are able to efficiently use the N lately applied in GS37 (Fuertes-Mendizábal et al., 2010b, 2013). The physiological basis of this agronomic behavior is based on the fact that in wheat, grain N is not only determined by the amount of N reserves accumulated in the vegetative organs during pre-anthesis, but also largely determined by the amount of N taken after anthesis, since both N sources contribute to storage protein accumulation in the grain (Dupont and Altenbach, 2003). In order to design N fertilization schedules tailored to a specific agroclimatic region, it is mandatory to get a better knowledge of the NUE by identifying the critical moments of requirements of N fertilizer by the crop during vegetative stages and those of N reallocation from source to sink organs.

An interesting tool to understand and interpret the efficiency of the different N fertilization regimes is the use of isotopic markers. The use of stable isotopes, particularly the analysis of N isotope natural abundance (δ^15^N), is promising in agriculture. This technique has the advantage that it is relatively accessible, as it can be measured in different plant organs or growth stages without any extra design in the trials and at a reasonable price.

The patterns of δ^15^N in the plant integrate, on one side, processes occurring in the soil (N cycling and loss), reflecting the isotopic fractionation during transformations of soil N; at the same time, δ^15^N represents an indicator of which source, soil vs. fertilizer, is the main one for the plant. Specifically in agricultural soils, soil δ^15^N signature is strongly affected by that of the N fertilizer (Choi et al., 2017); the large difference in δ^15^N among soil and N fertilizer allows the usage of ^15^N isotope composition as a tracer. Synthetic fertilizers are normally depleted or enriched in ^15^N. On the other side, plant values of δ^15^N also reflect the different physiological mechanisms occurring inside the entire plant system, mainly N uptake, nitrate reduction and N assimilation at different crop stages, as well as remobilization to the grain, or even N losses to the atmosphere or rhizosphere (Coomstock, 2001; Evans, 2001). Thus, the application of different N fertilizer doses and in different moments to match the N requirements of the crop along the growing season is foreseeable to influence the ^15^N plant composition, thus reflecting the N use by the plant. Under this assumption, the time-integrated character regarding biological processes makes δ^15^N is considered as a potential tool to be taken into account in order to address the understanding of the processes responsible for NUE under different climatic and agronomic management conditions. This technique has been used, for instance, to understand the ecosystem N status (Liu et al., 2017), as indicator of the fertilization regime (Bateman et al., 2005; Inázio et al., 2015; Choi et al., 2017), as a potential tool to guarantee the authenticity of organic products (Flores et al., 2007) or for estimating the relative contribution of the different N sources to a sink (Kolb and Evans, 2003; Serret et al., 2008; Flores et al., 2010). Nevertheless, the use of ^15^N natural abundance still demands of a deeper theoretical basis and technical advances in order to be correctly interpreted in the soil-plant system (Robinson, 2001), according to the farming system or agricultural practices.

The present work has been carried out under the following hypothesis: firstly, the δ^15^N in wheat is expected to deplete with increasing application of synthetic nitrogen fertilizer (Austin and Jones, 1975; Bateman et al., 2005; Bol et al., 2005), secondly, the third late amendment allows to increase the N content in grain in some wheat varieties (Fuertes-Mendizábal et al., 2010b, 2013). The specific objectives of the present work were: firstly, to explore whether the natural abundance of N isotopes technique could be used to understand the movement of N within the plant during vegetative and grain filling phases in wheat crop and, secondly, to evaluate the potentiality of δ^15^N value in tissues as a physioagronomic indicator to assess the effect of different fertilizer managements on NUE by wheat. The work has been done under humid Mediterranean conditions in the Northern Spain. Results are discussed in relation with N content in plant organs as well as with agronomic and (breadmaking) quality parameters of wheat grain and will help to provide scientific basis for a rational fertilization.

## Materials and Methods

### Experimental Design

**FIGURE 1:**
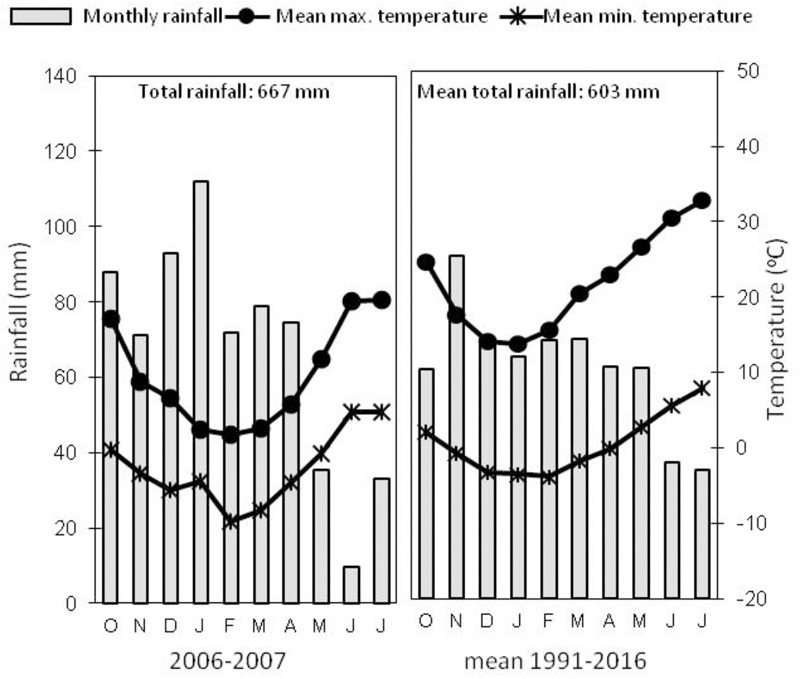
Mean monthly and total rainfall and minimum and maximum temperatures for the location at Arkaute (Alava, Spain) during the field trial in 2006–2007, and mean values for the last 25 years (1991–2016) (data source: www.euskalmet.euskadi.eus).

A field trial was established in Northern Spain in Arkaute (42°51′N, 2°41′W, and 513 m above sea level) in the province of Alava, on a clay loam soil, type Aquertic Etrudept (**Table 1**). The preceding crop was winter wheat. Mechanical tillage (disk, mouldboard plow) was used for seed bed preparation. Winter bread wheat (*Triticum aestivum* L.) Cezanne variety was sown at a density of 220 kg seeds ha^-1^ in November 2006 and harvested in July 2007. At pre-seeding 90 kg ha^-1^ of P_2_O_5_ and 90 kg ha^-1^ of K_2_O were applied as basal fertilization. The experimental design consisted in four N fertilizer treatments applied as ammonium nitrate (33.5% N w/w, with a δ^15^N value of 1.56 ± 0.17‰) at rates of 0, 140, and 180 kg N ha^-1^, splitted in two or three amendments at the beginning of tillering stage (GS20), the beginning of stem elongation stage (GS30) and flag leaf stage (GS37), as stated in **Table 2**. The trial consisted of a completely randomized block experimental design with four replications (number of total plots = 16), the size of each plot being 50 m^2^ (5 m × 10 m). Regarding the weather conditions, **Figure 1** shows monthly rainfall and temperatures at the trial location along the field campaign (2006–2007) and mean values of monthly rainfall and temperatures for the last 25 years (1991–2016). The rainfall during 2006–2007 growing season was slightly lower than the average values for the last 25 years, and temperatures fall within the region’s average annual.

**Table 1:** Physical and chemical properties of the clay loam soil (0–30 cm depth) for the location at Arkaute (Alava, Spain).

Soil texture			Soil chemical properties								
Sand (%)	Silt (%)	Clay (%)	pH	Organic matter (%)	N (%)	C/N	Carbonate (%)	P (ppm)	Ca (meq/100)	Mg (meq/100)	K (meq/100)
41.8	27.5	30.8	7.7	2.1	0.16	7.7	8.1	60.9	33.2	0.6	175.8

**Table 2:** Total rates and splitting of the different N-fertilization treatments, and soil and plant samplings on different growth stages (GS) along the crop cycle.

		GS20	GS30	GS36	GS37	GS60	GS90
	Treatments	Beginning of tillering	Beginning of stem elongation	Flag leaf stage		Anthesis	Maturity
Fertilization (kg N ha^-1^)							
	Test	0	0		–		
	140	40	100		–		
	180	40	140		–		
	180+	40	100		40		
Soil sampling				0–30 cm		0–30 cm	0–30 cm
Plant sampling				Flag leaf fourth leaf		Ear flag leaf fourth leaf	Grain ear flag leaf fourth leaf

*GS according to the Zadoks scale.*

### Sampling and Analyses

Soil was collected and wheat plants were harvested at different developmental stages. Soil was collected by mixing four subsamples taken individually from the 0–30 cm depth zone in each plot, just before the flag leaf (GS36), anthesis (GS60), and maturity (GS90) stages (**Table 2**). Fifteen wheat plants per treatment and plot were harvested and separated into fourth leaf (bottom leaf), flag leaf and ear, just before flag leaf (GS36), flowering (GS60), and maturity (GS90) stages. Values for mature grain corresponded to GS90 and were determined by harvesting the 1.5 m width central belt along each plot row. Both soil and plant samples were oven-dried at 70°C for 72 h and homogenized into fine powder for further analyses. In the case of the grain, the material was later sieved through a 1 mm screen. Breadmaking quality was determined using the standard alveogram procedure (AACC-Approved methods of the American Association of Cereal Chemists, 1993) and given as Chopin’s alveogram parameters: extensibility (L), tenacity (P), equilibrium (P/L), and strength (W). Nitrogen concentration and δ^15^N in soil and plant material were determined using a Delta Plus Continuous Flow Stable Isotope Ratio Mass Spectrometer (Thermo Finnigan, Bremen, Germany) coupled to a Carlo Erba elemental analyzer (CHNSO EA1108, Carlo Erba, Milan, Italy). Grain protein concentration was calculated as the product of total N concentration multiplied by 5.7 (Teller, 1932).

Nitrogen harvest index (NHI) was calculated accordingly to Austin and Jones (1975).

$$\text{NHI}\;=\;100(Y_{\text{G}}\cdot N_{\text{G}})/[(Y_{\text{G}}\cdot N_{\text{G}})\;+\;(Y_{\text{S}}\cdot N_{\text{S}})]$$

Where *Y* is the yield of grain (G) and straw (S), and *N* is the N concentration. The values of the isotope ratio were expressed as δ^15^N (‰), according to the formula

$$\delta^{15}\;N(‰)\;=\;[(R_{\text{sample}}/R_{\text{standard}})-1]\cdot 1000$$

where R_sample_ is ^15^N/^14^N of the soil or plant material. Following international convention, the R_standard_ used was the N isotope ratio in the atmosphere air (*R* = 0.0036765, ^15^N_air_ = 0‰_;_ Robinson, 2001). For ^15^N_air_, the samples were referenced against acetanilide. The δ^15^N of the material may be either positive if it is enriched in ^15^N relative to the standard, or negative if it is depleted.

### Statistical Analyses

Measurements were taken from four experimental replications. Data on N concentration and δ^15^N values were subjected to a two-factor ANOVA for soil data and three-factor ANOVA for plant data (SPSS version 20.0; SPSS Inc., Chicago, IL, United States). The variance was related to the three main treatments (fertilizer treatment, organ and sampling date) and to the interactions between them. Duncan’s multiple-range test was used to evaluate significantly different means (*P* < 0.05) between treatments.

## Results

### Grain Yield, Protein Content, and Breadmaking Quality Parameters

Wheat grain yield ranged from 3.20 tons/ha in the non-fertilized plants to 5.64 tons/ha in treatment 180+ (**Table 3**). The three-time splitted 180 kg N ha^-1^ rate maintained the yield as the same rate twice splitted. The non-fertilized plants showed a grain protein concentration of 7.3%, which increased to 10.3% and 10.9% by applying 140 and 180 kg N ha^-1^ two-times splitted, respectively, with no significant difference between them. However, the third splitting of the highest rate of 180 kg N ha^-1^ at GS37 led to a further increase in grain protein concentration of 7.8% respect to the same twice-splitted dose (**Table 3**). Besides, this increase in grain protein content due to the third split (180+) was accompanied by a significant increase (10%) in the NHI. The most determinant parameters of breadmaking quality, extensibility (L) and strength (W), were increased by 103% and 143%, respectively, on average when applying 140 and 180 kg N ha^-1^ (**Table 3**) with respect to the non-fertilized treatment. A further improvement in the dough strength (24%) occurred when the highest fertilizer dose was three times splitted in compared to treatment receiving two fertilizer amendments.

**Table 3:** Grain yield, grain protein content, Nitrogen Harvest Index (NHI) and breadmaking quality of dough, according to the Chopin’s alveogram parameters in wheat (L: extensibility; P: tenacity; P/L: tenacity/extensibility ratio or equilibrium; W: strength).

Kg N ha^-1^	Yield (kg ha^-1^)	Grain protein (%)	NHI (%)	Alveogram parameters			
				L (mm)	P (mm)	P/L	W (×10^-4^ J)
*Test* (0)	3196 ± 235 b	7.30 ± 0.11 c	78.3 ab	37.0 ± 2.3 b	57.7 ± 4.9 b	1.58 ± 0.17 b	87.7 ± 8.9 c
140	5441 ± 160 a	10.32 ± 0.28 b	79.4 ab	72.7 ± 7.7 a	86.2 ± 3.7 a	1.24 ± 0.18 a	216.3 ± 1.9 b
180	5412 ± 198 a	10.94 ± 0.45 b	74.5 b	78.0 ± 1.1 a	84.0 ± 4.0 a	1.03 ± 0.05 a	210.5 ± 0.3 b
180+	5639 ± 72 a	11.80 ± 0.17 a	81.8 a	93.0 ± 11.9 a	90.7 ± 9.6 a	1.07 ± 0.27 a	261.7 ± 4.8 a

*Nitrogen fertilization treatments: 0 (test), 140, 180, and 180+ (kg N ha^-*1*^) dispensed as indicated in **Table 2**. Values in the same column followed by the same letter are not significantly different at *p* < 0.05.*

### Soil N Content and δ^15^N

**FIGURE 2:**
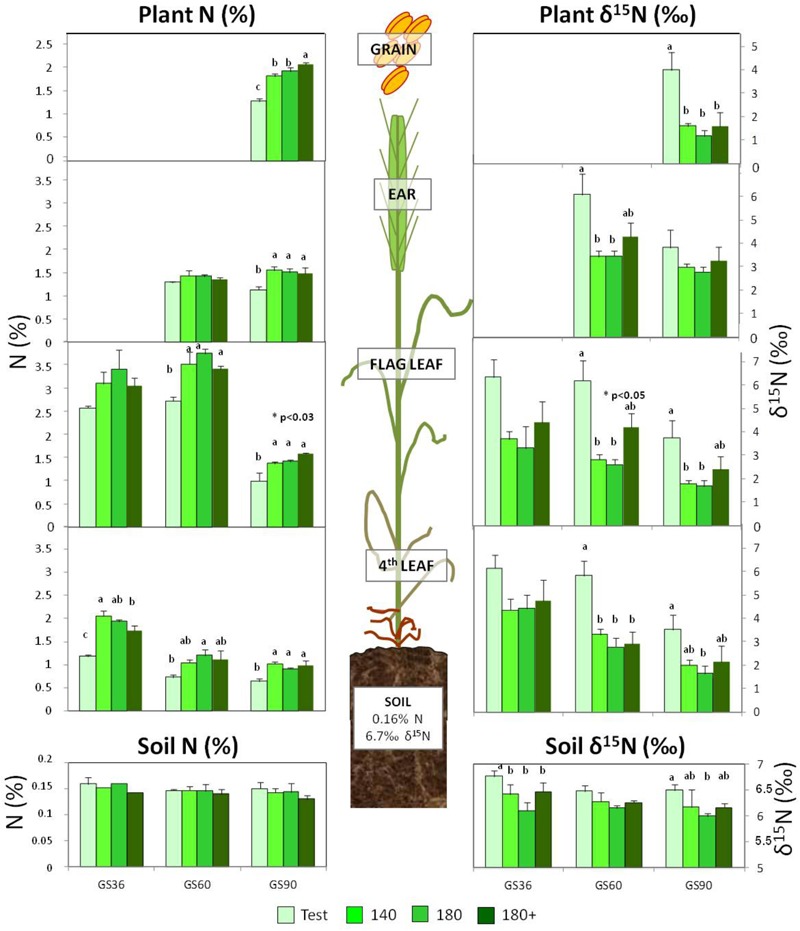
Effect of N fertilization on the N concentration and N isotopic composition (δ^15^N) of soil and plant organs at different growth stages. Fertilization treatments: 0, 140, 180, and 180+ (kg N ha^-1^) dispensed as indicated in **Table 2**. For each growth stage, different letters indicate significant differences between treatments at *p* < 0.05 according to Duncan’s test. Absence of letters indicates no differences. Asterisk indicates significant differences between treatments 180 and 180+, according to the *t*-test.

Soil N content remained stable independently of the fertilizer treatment along growth stages (**Table 4A**). The fertilizer applied (ammonium nitrate 33.5%) showed a low δ^15^N value of 1.56 ± 0.17‰, accordingly to the range of -1.4 to 2.6‰ described for NH_4_NO_3_ (Bateman and Kelly, 2007). Thus, the application of inorganic N fertilizer significantly decreased the soil δ^15^N in all fertilized treatments (**Figure 2**) at GS36 compared to the non-fertilized soil, which showed a δ^15^N value of 6.7‰. The soil δ^15^N remained stable at the different wheat growth stages (**Table 4A**).

**Table 4:** Summary of Factorial ANOVA.

(A) Soil factorial ANOVA				
	**Soil N (%)**		**Soil δ^15^N (‰)**	
	***F***	**Significance level**	***F***	**Significance level**

Treatment	1.351	ns	5.384	^∗∗^
Growth stage	1.302	ns	2.840	ns
Treatment × growth stage	0.125	ns	0.222	ns

**(B) Plant organs factorial ANOVA**				

	**Plant N (%)**		**Plant δ^15^N (‰)**	
Treatment	36.051	^∗∗∗^	31.697	^∗∗∗^
Growth stage	178.799	^∗∗∗^	33.595	^∗∗∗^
Organ	241.727	^∗∗∗^	3.27	^∗∗^
Treatment × growth stage	1.383	ns	0.981	ns
Treatment × organ	1.26	ns	0.742	ns
Growth stage × organ	106.738	^∗∗∗^	0.557	ns
Treatment × growth stage × organ	1.114	ns	0.187	ns

***(A)** Two-way ANOVA for the effect of N fertilization treatment and growth stage sampled on soil N content and δ^*15*^N. **(B)** Three-way ANOVA for the effect of the N fertilizer treatment, growth stage and organ on plant N concentration and δ^*15*^N. Treatments as in **Table 2**. Asterisks indicate significance level at ^∗∗^*p* < 0.05 and ^∗∗∗^*p* < 0.001.*

### Plant N Content and δ^15^N

Plant N concentration significantly changed with fertilizer application, along growth stages and among organs in wheat (**Table 4B**). In addition, an interaction between the growth stage and the organ was also observed. The δ^15^N values also showed differences with the fertilized treatments, growth stages and organs, without any interaction between these factors (**Table 4B**).

The N concentration of vegetative organs (fourth and flag leaves) showed a trend to diminish along the wheat crop lifecycle (**Figure 2**). Concomitantly, a N remobilization toward the grain occurred. Thus, an average decrease of 40% in the N concentration of the fourth leaf was observed between GS36 and GS60 in all treatments as a whole, while the flag leaf showed a higher decrease later (60%), from anthesis at GS60 to maturity at GS90. During this period, when it’s well known that the ear actively accumulates biomass, the N concentration remained stable. Regarding the effect of fertilization, the N concentration of vegetative organs increased in fertilized respect to non-fertilized plants, although no differences were found among fertilized treatments within the different stages. However, at harvest, grain N concentration was significantly increased with the three-time splitting (**Figure 2**).

Concerning the ^15^N isotope composition, as observed by other authors (Kolb and Evans, 2003) a decreasing trend of δ^15^N values within the different plant organs was observed as lifecycle draws on, decreasing in the non-fertilized plant organs from a mean 6‰ at GS36 and GS60 to 3.5‰ at GS90 and from a mean 4‰ to 2‰ in the fertilized plants (**Figure 2**). The δ^15^N value of the grain in fertilized plants was significantly lower than their respective leaf and ear values (**Figure 2**).

Nitrogen fertilization induced changes in δ^15^N values, leading to enrichment in the heavy ^15^N isotope in non-fertilized plants (average δ^15^N value of 5‰) with respect to the fertilized plants (average δ^15^N value of 2.9‰), whatever the organ sampled (**Figure 2**). Increasing the N rate from 140 to 180 kg N ha^-1^, but maintaining the splitting at the same moments (GS20 and GS30) led to equal plant natural N isotopic composition (**Figure 2**). On the contrary, a late splitting of the 180 kg N ha^-1^ rate (three-times splitted) raised δ^15^N in the flag leaf at GS60 and GS90 and in the ear at GS90 with respect to the two-time splitting.

## Discussion

### Further Splitting the N Rate Is Effective in Maintaining Wheat Yield and Improving Grain Quality Under Humid Mediterranean Conditions

The right rate and timing of fertilizer application must meet the nutrient crop necessity in order to optimize the plant N uptake and avoid N losses to the environment. In this sense, a third late N-fertilizer application in wheat crop at GS37 has been proposed as an agronomic management aimed to increase the NUE by the crop; and this application results particularly interesting in our region (Fuertes-Mendizábal et al., 2010a), with a monthly average rainfall of 70 mm from March to May in the last 25 years (**Figure 1**). In fact, when there is no water limitation this late amendment is able to raise the photosynthetic capacity and the carbohydrate accumulation in leaves and to stimulate the wheat canopy development during the vegetative stages; thus, achieving higher grain yields in Soissons wheat variety (Fuertes-Mendizábal et al., 2010b) and higher grain protein contents in Cezanne variety (Fuertes-Mendizábal et al., 2013). The present experiment confirms that, the rainy spring of our region allows the incorporation of the N supplied lately at GS37 (in May) in Cezanne variety. Actually, similarly to what happens in other regions of Northern Europe, further splitting of the N dose at the flag leaf stage (GS37) maintains the crop yield, what means it can correct a possible decrease in yield because of a limiting second application of only 100 kg N ha^-1^ at the beginning of stem elongation (GS30).

Regarding grain quality, it is widely known that the increment in grain protein content is usually translated into a better breadmaking quality of the flour. In this sense, dough strength (W) and extensibility (L) have been described as the most sensitive alveogram parameters to N management and the most dependent properties on grain protein content (Ames et al., 2003). Thus, under Mediterranean climate conditions, Garrido-Lestache et al. (2005) described improvements in grain quality after the splitting of the N rate, although this effect has not been clearly stated in other studies (Ayoub et al., 1994). In our study the increment in grain protein content by three-time splitting of the same N rate (180 Kg N ha^-1^) was translated into an improvement of the dough strength of Cezanne variety (**Table 3**). According to Borghi et al. (1995), the flours obtained from the fertilized treatments of the present experiment (W > 210. 10^-4^ J) can be classified inside class 2, e.g., suitable for “direct breadmaking quality.” A better behavior of W is reached, with values up to 261.7 10^-4^ J, when the fertilizer is three-times splitted, which categorizes this flour into class 1 and enables its use as an “improver wheat.” Whereas on the contrary, when no fertilizer was applied the low values of W and L makes this flour is classified as “bread for biscuit,” with a quality not appropriate for breadmaking. So, we conclude that, similarly to what was described for Soissons variety under the same climatic conditions by Fuertes-Mendizábal et al. (2010a, 2012), the management of N fertilization through a third late splitting at GS37 also improves grain quality in Cezanne variety destined to breadmaking.

### Fertilization Depletes the Soil δ^15^N

The soil is considered as a dynamic system where many N transformation processes occur, as mineralization, immobilization, nitrification, volatilization, or plant assimilation. All these processes are viewed as reciprocal and, taken as a whole, they would have none or little effect on total soil N and δ^15^N. Total soil N and δ^15^N are dominated by soil stable N (Johannisson and Högberg, 1994) and by both N inputs and outputs fluxes (Liu et al., 2017); possible changes in these parameters would be originated from the N input–output balance in the soil system, such as fertilizer application or N losses to the environment. Provided different studies show that mineral fertilization can either decrease (Serret et al., 2008), increase (Senbayram et al., 2008; Liu et al., 2017) or even have none (Choi et al., 2003; Kriszan et al., 2009) or little effect (Chen et al., 2011) on soil δ^15^N, in our study, we monitored the soil N content in combination with soil δ^15^N to attain a better interpretation of possible variations in the N source available for the plant due to fertilizer management. The soil of our study presented a N content of 0.16% previous to N fertilization (**Table 1**), and was not affected by the input of mineral N fertilizer (**Figure 2** and **Table 4A**). In fact, the only expected changes in soil “N fractions” after mineral fertilization would be a transient increase in soil ammonium and nitrate contents (Huérfano et al., 2016), whereas no clear dependence of top-soil N content and the amount of applied fertilizer is expected (Watzka et al., 2006). Regarding soil δ^15^N, the initial value of 6.7‰ (**Figure 2**) is in the range of cultivated soils (+1 and +12‰) (Choi et al., 2017), which is normally higher than in natural soils due to the fertilization input. Thus, the δ^15^N of cultivated soils is susceptible to be affected by the δ^15^N of the fertilizer after long periods applying N. In the same line of evidences, soil δ^15^N values even below 0‰ (Broadbent et al., 1980) and a strong depletion in soil δ^15^N, with a lowering of 2.9‰ (Gerzabek et al., 1999) have been registered. The slight downward shift of 0.31‰ units observed after fertilization in our short-time study would be explained by the low signature value of the mineral nitrogen fertilizer applied (Choi et al., 2017), which showed a δ^15^N value of 1.56 ± 0.17‰, e.g., close to that of atmospheric N_2_ (0‰).

More interestingly, the lowering shift effect of δ^15^N was distinguishable among the different fertilization strategies in GS36, just before the application of the third amendment. Thus, the impoverishment trend in the soil δ^15^N value observed in the treatment 180 reflected that the amount of fertilizer dispensed until that very moment was the highest rate (**Table 2** and **Figure 2**). Treatments 140 and 180+ showed identical soil δ^15^N values, accordingly to the fact that both plots had received equal N fertilizer rate and timing (40 kg ha^-1^ at GS 20 and 100 kg ha^-1^ at GS30).

### Plant δ^15^N Reflects the N Source Signature and Shows Isotope Discrimination During N Remobilization Toward the Grain

The interpretation of ^15^N natural abundance in plant tissues is generally complex because δ^15^N signatures can differ from those of the original source due to isotope fractionation during physiological processes within the plant, mainly during N uptake, nitrate reduction and assimilation (Evans et al., 1996; Robinson, 2001), as well as during remobilization to the grain. Therefore, the combination of δ^15^N signature with other agrophysiological parameters becomes necessary to better understanding of the N movement within the plant, as modulated by the different fertilizer management strategies.

During pre-anthesis, up to GS60, leaves act overall as photosynthetic N sinks where N uptake and assimilation take place. It is during this phase when the wheat crop assimilates most of its N, in such a way the total N content in the plant at GS60 becomes sometimes as high as 90% of the total N at maturity (Heitholt et al., 1990; Hocking and Staper, 2001). Between GS36 and GS60, the fourth leaf strongly contributes to N remobilization, as indicated by its depletion in N content (**Figure 2**); Meanwhile, the flag leaf would still be growing (Gustavsson, 2011) and maintaining its assimilatory capacity, acting thus overall as a photosynthetic N sink. At GS60 the ear is completely developed (Zadoks et al., 1974) and can take up N directly from the soil, but it also behaves as a sink receiving N remobilized from leaves (Kichey et al., 2007). The lower δ^15^N values in leaves of the fertilized plants during pre-anthesis (**Figure 2**) indicate that more N was available for the plant directly from the fertilizer applied. In fact, the always higher δ^15^N values in organs of non-fertilized plants (with mean values of around 6.2‰) mirror more closely the soil δ^15^N value (with mean values of around 6.6‰), whereas fertilized plants show a mean drop of 2.5‰ units in the δ^15^N values respect to fertilized soils (with values between 3 and 4‰ with respect to 6.324‰ of the soil). This impoverishment in the heavy isotope in fertilized plants is interpreted in the sense that they were fed by a mixed source consisting of both soil N (with a δ^15^N value of 6.324‰) and the inorganic N fertilizer (with a low δ^15^N value of 1.56‰). Discrimination against the heavy ^15^N isotope occurs during the primary assimilation of inorganic nitrogen within the plant, nitrate reductase and glutamine synthetase being the main enzymes responsible for this isotope discrimination (Kalcsits et al., 2014). So, the impoverishment in δ^15^N of the fertilized plants induced by the uptake of inorganic fertilizer (**Figure 2**) was surely enhanced by an also higher N assimilation rate with respect to the non-fertilized plants.

During the grain filling period, from GS60 to GS90, the ear is the main sink organ and vegetative organs behave as sources remobilizing N in a type of senescence process characterized by a sequential death from bottom-to-upper leaves. In fact, this senescence process was formerly started in the pre-anthesis phase in the oldest fourth leaf. From GS60 to GS90 the contribution of the already senescent fourth leaf to N remobilization was minimal, upper leaves taking their turn successively jointly with leaf sheaths or even root, to supply N reserves to the ear (Fuertes-Mendizábal et al., 2012). Thus, it would be the youngest flag leaf the one providing N reserves for N recycling, as indicated by the strong depletion of more than 50% in its N content from GS60 to GS90 (**Figure 2**). Therefore, during the post-anthesis period it is the ear the plant organ that accumulates N. However, a dilution effect due to its continuous growth would be the responsible factor for counteracting the expected increase in N content due to the import of nitrogenous compounds, thus making the ear N concentration remains unchanged until maturity.

As leaves suffer the source-sink transition, ammonium is released from protein degradation (Kichey et al., 2006; Bernard and Habash, 2009). In theory, the lighter isotope ^14^N is often incorporated for the biosynthesis of the products destined to translocation (e.g., amino acids), whereas the heavier ^15^N isotope is concentrated in the remaining cell solution, since ^15^N-bearing molecules or ions react slower than isotopically lighter analogs (Dawson and Brooks, 2001). According to that, we would expect that the remaining leaves as source organs were enriched in ^15^N-bearing molecules non-used for remobilization. But interestingly, the remobilization of N from the fourth and flag leaves was concurrent with a significant impoverishment in the heavy ^15^N isotope as leaves senesced. Related to this, a general isotopic impoverishment of plant organs along the plant lifecycle was previously reported (Flores et al., 2007), although the physiological basis remains to be understood. The cytosolic isoform of glutamine synthetase (GS1) has been specifically involved in the reassimilation of ammonium released during senescence. Kichey et al. (2006) reported its accumulation in the mesophyll cytosol in the flag leaf. Glutamate dehydrogenase is also induced in senescent leaves probably to deaminate leaf proteins (Kichey et al., 2005). Whatever the enzyme is contributing to the recycling of ammonium, a discrimination effect against ^15^NH_4_^+^ occurs, the lighter ^14^NH_4_^+^ being preferentially incorporated by N-reassimilating enzymes for amino acid translocation. According to that, we suggest that ammonium peaks occurring in senescing leaves will be proportionally ^15^N-enriched. Several authors have reported that ammonia emissions peak related to N remobilization take place during late leaf senescence (Wang et al., 2011; Wang and Schjoerring, 2012). Thus, the impoverishment in ^15^N observed in senescent organ biomass might be explained by ^15^N-enriched ammonia losses from the plant body to the atmosphere when N remobilization occurs.

**FIGURE 3:**
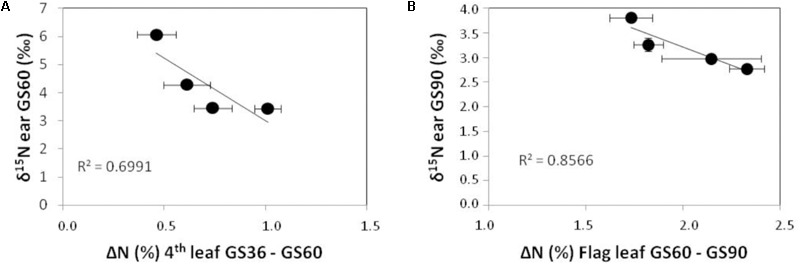
Correlation between ear δ^15^N values at GS60 and decreases in the fourth leaf N concentration from GS36 to GS60 **(A)**; and between ear δ^15^N values at GS90 and decreases in the flag leaf N concentration from GS60 to GS90 **(B)**.

The dynamics of δ^15^N values in the ear must be interpreted in function of the dual role of this structure both as sink and source of N for remobilization. The ear δ^15^N values are concomitant with changes in N contents of the leaves as N remobilizing organs. In fact, the strong correlations between the ear δ^15^N values at G60 and GS90 with changes in N concentration of fourth and flag leaves, respectively (**Figure 3**) support that fourth leaf provides the ear with N from G36 to GS60 whereas flag leaf provides more directly N to the ear from the anthesis (GS60) to maturity (GS90). On the other hand, the extremely low values of δ^15^N in the non-fertilized ear at GS90 respect to GS60 indicate that during grain filling the N previously carried-over from leaves is isotopically discriminated in the same ear structures, and also that the remobilization process would predominate over a direct N uptake from soil by this organ. The fact that grain δ^15^N signature of non-fertilized plants shows similar values to the ear and flag leaf at GS90 (around 4‰), indicates: firstly, the ear structures contribute to grain filling, playing an intermediate role during the development of mature grain. In fact, practically all the N remobilized is temporarily accumulated in the glumes, which act as a sink of N before its further remobilization to the grain (Lopes et al., 2006). In the same line of evidence, Sanchez-Bragado et al. (2016) have reported that ammonium turnover happens actively in different structures of the ear (glumes, awns, grains) as well as in the flag leaf. The remobilization process could explain the strong impoverishment in ^15^N observed in the non-fertilized ear between GS60 and GS90, as well as in the grain in fertilized plants (with δ^15^N of 1.7–2.0‰) in relation to their respective ears (δ^15^N of 2.6–3.2‰) or flag leaves at GS60 (δ^15^N of 2.8–4.2‰). Secondly, the discrimination against ^15^N is minimal in the non-fertilized ear compared to the fertilized one, surely due to the N limitation prevents the discrimination and promotes a simultaneously higher efficiency in the N remobilization process (**Figure 3**) (Gaju et al., 2014). The larger amount of N carried over to the grain, the higher the discrimination against ^15^N becomes in this structure. This fact is supported by the strong depletion in δ^15^N values for grain in fertilized plants regarding non-fertilized plants, which is not evident in the ear at GS90, and the improvement in N content in the fertilized treatments (**Figure 2**). And also by the inverse correlations observed between ear δ^15^N values and the changes in N concentration of vegetative organs acting as N source along the wheat crop cycle (**Figure 3**). Thus, we can interpret these relationships in the sense that discrimination within the plant is lower in the non-fertilized ear because the ratio between plant demand versus the N available is high (Serret et al., 2008).

A third late N fertilizer application at the flag leaf stage (GS37) attains a better behavior of dough as consequence of the increment in grain protein. Nevertheless, no effect of the third late amendment was evident in terms of N concentration in the ear, as was neither observed in general for the rest of vegetative organs along the crop cycle. According to previous work (Fuertes-Mendizábal et al., 2012), splitting the same dose into three amendments did not increase the N reserves in tissues but remobilize them more efficiently. The late N application would have more impact on N metabolism and its remobilization to the grain than on biomass accumulation or N partitioning in the plant (Fuertes-Mendizábal et al., 2010b). The δ^15^N signature reveals itself as a physioagronomic marker able to reflect the effect of a late N amendment in treatment 180+ in certain organs and moments. Thus, the δ^15^N in the flag leaf and the ear at GS60 allows tracking the effect of the application of this third N amendment. Besides, the higher δ^15^N values were determined in the flag leaf and ear in treatment 180+ at GS60, which confirms the period between GS36 and GS60 is critical for N uptake by the crop under humid Mediterranean conditions. Moreover, once the third amendment is dispensed it would be expected the plant uses preferably the N coming directly from the fertilizer, however the higher δ^15^N value of the flag leaf and ear at GS60 in treatment 180+ is also maintained respect to the values of the previous growth stage GS36. Interestingly, this indicates that the plant from treatment 180+ is taking proportionally a higher amount of N coming from the soil than in the case of 140 and 180 plants. In other words, the δ^15^N parameter allows to illustrate that after applying a late N amendment the plant takes up an extra quantity of N coming from soil at this late growth stage, which would contribute to the increase in grain N content and dough quality observed, thus implying a more efficient use of the N sources compared to a twice amendment management.

## Conclusion

In conclusion, the use of the nitrogen isotopic natural abundance technique in wheat plants revealed that N remobilization is a discriminating process leading to an impoverishment in the heavy isotope of senescent leaves and grain. The analysis of δ^15^N values also reflected how wheat plants are able to use nitrogen resources (native soil-N versus fertilizer-N) in a more efficient way when N application is further splitted into a late N amendment under humid Mediterranean conditions.

## Author Contributions

TF-M, JME, and MBG-M designed the experiments, developed the structure and arguments for the paper, made critical revisions, and had primary responsibility for the final content. AA and AC managed the field experiments. XH and MKD helped in analyzing the data. JME, CG-M, and MKD supervised the experiments and complemented the manuscript writing and its discussion. All authors have read and approved the final manuscript.

## Conflict of Interest Statement

The authors declare that the research was conducted in the absence of any commercial or financial relationships that could be construed as a potential conflict of interest.
